# Obesity Arrhythmias: Role of IL-6 Trans-Signaling

**DOI:** 10.3390/ijms25158407

**Published:** 2024-08-01

**Authors:** Kelly A. Aromolaran, Andrea Corbin, Ademuyiwa S. Aromolaran

**Affiliations:** 1 Nora Eccles Harrison Cardiovascular Research and Training Institute (CVRTI), University of Utah School of Medicine, Salt Lake City, UT 84112, USA; kelly.aromolaran@utah.edu (K.A.A.); andrea.corbin@utah.edu (A.C.); 2 Department of Biomedical Engineering, University of Utah School of Medicine, Salt Lake City, UT 84132, USA; 3 Department of Surgery, Division of Cardiothoracic Surgery, Nutrition & Integrative Physiology, Biochemistry & Molecular Medicine Program, University of Utah School of Medicine, Salt Lake City, UT 84132, USA

**Keywords:** IL-6 trans-signaling, cytokines, obesity, ion channels, ventricular arrhythmias

## Abstract

Obesity is a chronic disease that is rapidly increasing in prevalence and affects more than 600 million adults worldwide, and this figure is estimated to increase by at least double by 2030. In the United States, more than one-third of the adult population is either overweight or obese. The global obesity epidemic is a major risk factor for the development of life-threatening arrhythmias occurring in patients with long QT, particularly in conditions where multiple heart-rate-corrected QT-interval-prolonging mechanisms are simultaneously present. In obesity, excess dietary fat in adipose tissue stimulates the release of immunomodulatory cytokines such as interleukin (IL)-6, leading to a state of chronic inflammation in patients. Over the last decade, increasing evidence has been found to support IL-6 signaling as a powerful predictor of the severity of heart diseases and increased risk for ventricular arrhythmias. IL-6′s pro-inflammatory effects are mediated via trans-signaling and may represent a novel arrhythmogenic risk factor in obese hearts. The first selective inhibitor of IL-6 trans-signaling, olamkicept, has shown encouraging results in phase II clinical studies for inflammatory bowel disease. Nevertheless, the connection between IL-6 trans-signaling and obesity-linked ventricular arrhythmias remains unexplored. Therefore, understanding how IL-6 trans-signaling elicits a cellular pro-arrhythmic phenotype and its use as an anti-arrhythmic target in a model of obesity remain unmet clinical needs.

## 1. Introduction

Sudden cardiac death (SCD) is one of the most common causes of death and accounts for 180,000–300,000 events per year in the United States [1,2]. In nearly two-thirds of events, SCD is the result of malignant ventricular arrhythmias, such as ventricular tachycardia (VT) and ventricular fibrillation (VF) [3,4]. At the cellular level, prolongation of the ventricular action potential duration (APD), with a subsequent increase in early afterdepolarizations, underlies an elevated risk for prolonged QT-associated life-threatening ventricular arrhythmias [5]. Patients developing marked QT interval prolongation and fatal ventricular arrhythmias concomitantly present with multiple risk factors [6]. In one study of 40 consecutive unselected patients with torsades de pointes (TdP), >4 factors (electrolyte imbalances, cardiac and extracardiac diseases, drugs, anti-Ro/SSA antibodies, and inflammation) per subject were detected, with a high prevalence of acquired channelopathies [7]. Therefore, efforts to avoid or discontinue drugs that may exacerbate mechanisms that elevate the risk of SCD are increasing, particularly in patients with existing risk factors. 

Obesity increases ventricular arrhythmia risk [8,9,10,11], particularly under conditions of repolarization disorder and prolonged QT interval (an established risk factor for VT) [12,13], and is the most common non-ischemic cause of SCD [5,14,15]. According to 2017–2018 data from the National Health and Nutrition Examination Survey (NHANES) (https://www.cdc.gov/nchs/data/hestat/obesity-adult-17-18/obesity-adult.htm, accessed on 4 January 2023), nearly 1 in 3 adults (30.7%) are overweight, more than 2 in 5 adults (42.4%) have obesity, and ~1 in 11 adults (9.2%) have severe obesity [16]. In the Framingham Heart Study, after 26 years of follow-up, obese men had a 2.6-fold increased risk of SCD compared with lean individuals [17]. The impact of obesity was even more deleterious for women, with a 5.8-fold increase in SCD risk [17], which suggests differences in the pathogenesis of arrhythmia/SCD between men and women. Thus, with the increasing prevalence of obesity in populations with a longer life span, identifying and validating the cellular pro-arrhythmic mechanisms that elevate life-threatening ventricular arrhythmias/SCD risk in the setting of obesity is important. 

Obesity is a multifactorial chronic disease [18] and, therefore, may predispose to an increased risk of arrhythmia/SCD risk through various molecular mechanisms. Obesity-related heart diseases are generally attributed to co-existing structural diseases, particularly coronary artery disease (CAD) [19,20,21] and heart failure (HF) [22]. However, in a postmortem examination of SCD cases, structural alterations were not identified in 5–15% of all patients and in ~40% of young patients aged <40 years [23,24]. Thus, the need to identify the non-structural obesity mechanisms that contribute to increased arrhythmias/SCD risk remains unmet. 

Recapitulating most of the clinical features of human obesity or dissecting the unique mechanistic contribution of obesity relative to the associated features of metabolic disorders (including type 2 diabetes) [25,26] toward arrhythmia/SCD risk in preclinical animal models may not be feasible. However, an important objective may be to identify a common and emerging cellular pathology. Lipids are a critical and independent predictor of metabolic disorders, obesity [25,26], and related cardiac dysfunction [27,28,29,30,31]. This relates to the abnormal accumulation of cardiotoxic free fatty acids (FFAs or lipotoxicity) and the subsequent activation of inflammation and alterations in pro-inflammatory cytokines. We suspect that inflammatory heart disease may exist, both alone and in concert with the well-recognized causes (CAD and HF) of heart disease, and impair the ability of the heart to compensate for them. In the past decade, the pro-inflammatory cytokine interleukin (IL)-6′s classical and trans-signaling pathways have emerged as powerful predictors of HF and VT risk [32,33,34]. However, the prognostic role of overexpressed cytokines [35] or the therapeutic potential of anti-cytokine drugs in obesity and associated ventricular arrhythmias is unknown. Without such knowledge, the realization of potential therapeutic targets for this dangerous arrhythmia will remain unfulfilled. 

## 2. IL-6 Signaling

**Figure 1:**
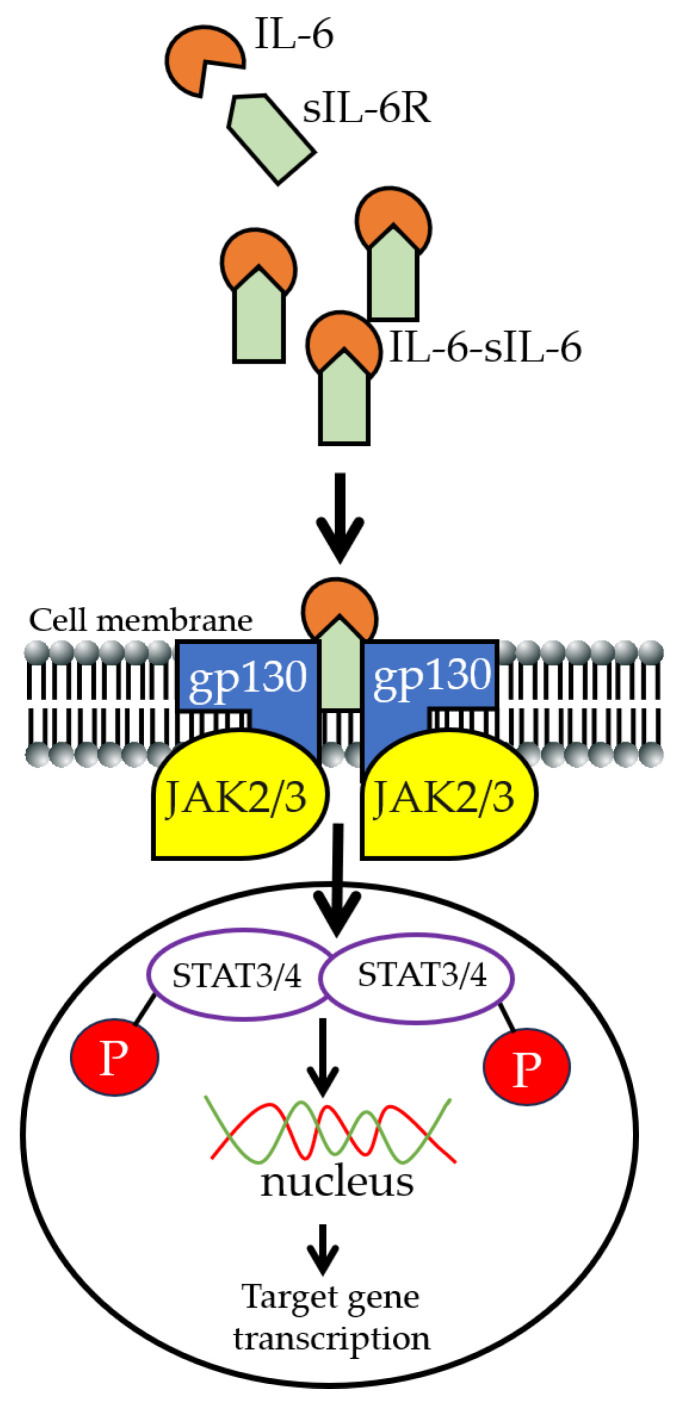
Cartoon illustration highlighting the well-established IL-6 trans-signaling pro-inflammatory pathway [45] leading to the activation of downstream JAK-STAT [43,46,47,48]. The potential therapeutic benefits and efficacy of selectively targeting IL-6 trans-signaling for the prevention of dangerous ventricular arrhythmias in obesity are unknown. STATs, upon phosphorylation, dimerize and translocate to the nucleus, where they can induce the expression of genes involved in proliferation and differentiation [51,52,53,54].

IL-6 is a pleiotropic cytokine (downstream from IL-1β) [36] that is involved in a variety of biological effects (both regenerative and pro-inflammatory) [37,38,39]. Classical IL-6 signaling occurs through its membrane-bound receptor (IL-6Rα), glycoprotein 130 (gp130 receptor complex), and mediates homeostasis and regenerative functions [37]. The soluble IL-6 receptor (sIL-6R) is generated by extracellular shedding or alternative processing of the mRNA encoding IL-6R [40]. IL-6 pro-inflammatory effects are mediated via trans-signaling [41,42] and might provide another alternative to steroidal drugs, with increased efficacy and decreased side effects in inflammatory diseases. IL-6 trans-signaling is activated through IL-6 binding to the sIL-6R [43,44], and this, in turn, engages gp130 on target cells [45], leading to the activation of downstream Janus kinase/signal transducers and activators of transcription (JAK-STAT) [43,46,47,48]. Anti-IL-6 trans-signaling pathway inhibitors are thought to provide another alternative to steroidal drugs, with increased efficacy and decreased side effects. An important feature of the IL-6 trans-signaling pathway is the ability of the IL-6/sIL-6 complex to bind gp130 (Figure 1), which is robustly and widely expressed in a variety of cell types [41,42]. Therefore, all cells of the cardiovascular system can be activated by the IL-6/sIL-6R complex, which further highlights the complexity of therapeutically targeting IL-6 trans-signaling. The hyper-IL-6 protein pioneered by Dr. Stefan Rose-John, which consists of the covalently linked bioactive parts of IL-6 and the sIL-6R α subunit [49], allows for the selective activation of IL-6 trans-signaling without any classical signaling activation and is now widely used to investigate the functional outcome of overactivated IL-6 trans-signaling. Another selective inhibitor of IL-6 trans-signaling is sgp130Fc (a soluble gp130Fc-fused chimera), an optimized fusion protein of the natural sgp130 and IgG1-Fc [50]. Mechanistically, sgp130Fc binds to IL-6/sIL-6R complexes without any measurable effects on IL-6 alone or on the cell membrane IL-6R. Compared with endogenous sgp130, sgp130Fc possesses about 10 to 100 times greater ability to inhibit IL-6 trans-signaling pathological effects. Notably, the sgp130Fc fusion protein is in clinical trials for the treatment of several inflammatory diseases [50]. The continued use of selective inhibitors of IL-6 trans-signaling together with global IL-6 inhibitors is likely to improve our current understanding of IL-6 signaling, with implications for improved and effective therapeutic interventions in patients with acquired inflammatory diseases and arrhythmias.

Although IL-6 is known to play a prominent role in the pathogenesis and prognosis of ventricular electrical remodeling [33,34] and in the future development of ventricular arrhythmia events, the mechanisms underlying its action on cardiomyocytes and in the heart are relatively unexplored. 

Previous studies have discovered that lipotoxicity is associated with an altered expression of JAK2 [55,56,57], suggesting its involvement in ventricular arrhythmogenesis. Although other studies have investigated how JAK2 modulates the Na_v_1.6 channels in hippocampal CA1 pyramidal neurons [58], the K_v_1.3 channels in CHO-cells [59], the BK [60], CIC-2 [61], and epithelial Na channels [62] in *Xenopus oocytes*, and the Ca current in MCF-7 breast carcinoma cells [63], how JAK2 modulates the rapidly activating delayed rectifier K current (*I_Kr_*) in cardiomyocytes remains unknown. 

We have identified a previously unrecognized link between JAK2, *I_Kr_* dysfunction, and ventricular APD remodeling [33]. Specifically, we have found that the JAK2 inhibitor AG490 prevents decreases in *I_Kr_*, APD prolongation, and the rightward shift in the voltage-dependent activation of *I_Kr_* due to the exogenous application of IL-6 and sIL-6R in freshly isolated guinea pig ventricular myocytes. Thus, the activation of JAK2 raises the intriguing possibility that the modulation of *I_Kr_* by IL-6 depends on tyrosine phosphorylation. However, whether and how JAK2 phosphorylates human ether-á-go-go-related (hERG) channel subunits in the heart remains unclear. Protein kinase phosphorylation regulates *I_Kr_*, significantly reducing currents [64,65,66,67], while constitutively active protein tyrosine kinase (PTK) c-Src (Y530F) depresses currents and right-shifts the activation voltage of hERG currents in HEK293 cells [68]. Thus, future phosphoproteomics studies will likely allow for the identification of potential JAK2 phosphorylation sites on the hERG channel and provide the first mechanistic studies of how JAK2 affects the processes that regulate *I_Kr_* channel function. 

STATs are downstream from JAKs and, upon phosphorylation, dimerize and translocate to the nucleus, where they can induce the expression of genes involved in proliferation and differentiation [51,52,53,54]. JAK2-STAT3 contributes to an up-regulation of Na channels in DRG neurons [69] and an IL-6-mediated inhibition of the inward TRPM7 currents in both primary cultured neurons and HEK293 cells [70]; however, how JAK/STAT regulates *I_Kr_* remains unknown. Specifically, no extant studies have examined the role of JAK2-STAT3/4 in *I_Kr_* regulation in the heart, which could, therefore, represent a novel anti-arrhythmia target.

Intriguingly, evidence shows that STAT4 is involved in cardiovascular diseases [71,72,73]. However, the role of STAT4 in pathological IL-6 trans-signaling effects is unknown. A likely mechanism is through the downstream epigenetic regulator histone deacetylase (HDAC). Reports have shown HDAC involvement in pro-inflammatory responses [74,75] and inhibition by STAT4 [76]. Moreover, the cardiac-specific deletion of HDAC3 resulted in cardiac hypertrophy and the upregulation of genes associated with fatty acid uptake, fatty acid oxidation, and electron transport/oxidative phosphorylation, which was accompanied by fatty-acid-induced cardiac lipid accumulation and elevated triglyceride levels [77]. Furthermore, cardiac and skeletal HDAC3 inactivation (postnatally) induced severe hypertrophic cardiomyopathy, HF, and death in high-fat diet (HFD)-fed mice [78], highlighting a critical and potential role for STAT-linked HDAC in arrhythmia risk with obesity. Thus, in contrast to the reported IL-6 blocker Tocilizumab (TCZ)-mediated lipid accumulation manifested as off-target effects, HDAC3 activators may prevent cardiac lipid accumulation, the subsequent activation of inflammatory pathways, and ultimately, IL-6-mediated adverse ventricular electrical remodeling. 

## 3. IL-6 and Ventricular Electrical Activity

**Figure 2:**
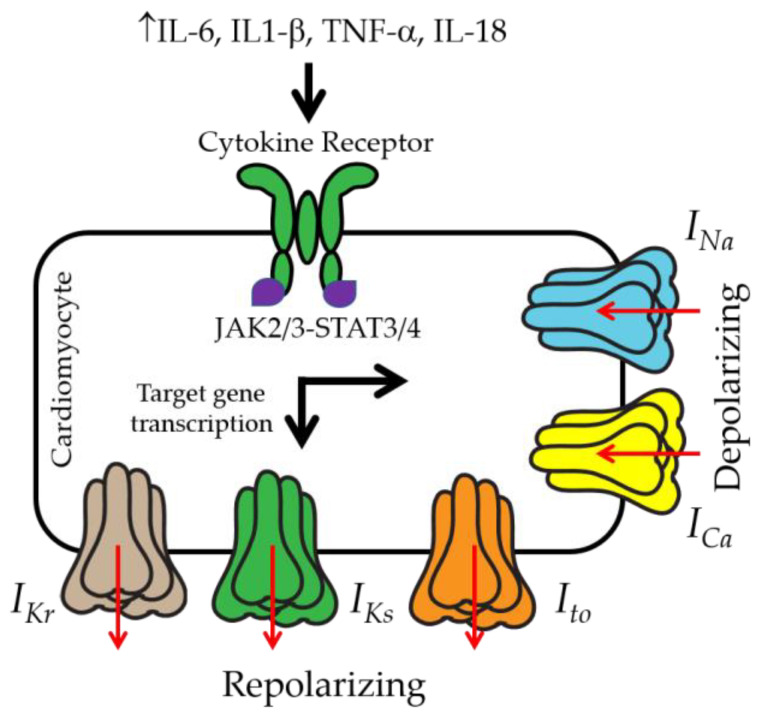
Cartoon representation of major ventricular depolarizing and repolarizing mechanisms that are prime candidates for modulation by individual and/or multiple combinations of pro-inflammatory cytokines pathologically elevated in obesity and associated pathologies (diabetes, inflammation, and hyperlipidemia). However, little work has been completed to date on mechanisms by which overexuberant inflammation and pro-inflammatory cytokine production can exacerbate pathologic ventricular electrical remodeling in obesity-linked ventricular arrhythmias. We suspect that overactivated cytokine-mediated remodeling processes may occur through the altered gene and protein expression of ion channel subunits and biophysical defects (subunit trafficking and/or gating defects). We hypothesize that understanding the contribution of distinct cellular arrhythmia triggers sensitive to cytokine mechanisms is important in interpreting the mechanistic bases of how cytokine channelopathies promote arrhythmia/SCD risk and informing on targeted anti-arrhythmia therapy in patients.

Inflammatory pathways are directly linked to cellular and subcellular signaling mechanisms that are known to provoke ventricular arrhythmias [79]. Among these, ion channel dysfunction remains one of the crucial factors in ventricular arrhythmia initiation. The normal ventricular cardiac action potential (AP) is defined as follows: Phase 0 is due to a large inward sodium current (*I_Na_*). Phase 1 is defined by the inactivation of the voltage-gated Na channels and the activation of the transient outward K current (*I*_to_). Phase 2, or the plateau phase, is maintained by currents due to voltage-gated L-type Ca (*I_Ca_*_,*L*_) and Na–Ca exchanger (*I_NCX_*) channels [80,81]. Repolarization (Phase 3) is controlled by a delayed rectifier current (*I_K_*), comprising the rapid (*I_Kr_*) and slow (*I_Ks_*) components. The resting membrane potential is controlled by the inward rectifier *K* current (*I_K_*_1_) [82]. Thus, decreases in repolarizing currents [83,84] or increases in depolarizing mechanisms [85] delay repolarization, resulting in prolongation of a heart-rate-corrected QT interval (QT_c_), which predisposes to fatal ventricular arrhythmias such as TdP [86] and SCD [87,88]. Most cases of clinically relevant acquired QT interval prolongation occur via the inhibition of *I_Kr_* [89]. A QT interval and the reversal of inflammation-driven QT changes directly correlate with cytokine levels [90,91,92,93] and further underscore the direct functional effect of cytokines on cardiac electrophysiological properties (Figure 2). 

Reports have shown that 24.3% of obese patients have prolonged QT_c_ intervals compared with patients with normal weight [94,95]. IL-6 [96] increases *I_Ca_*_,*L*_ (which increases calcium load in myocytes and can be associated with ventricular arrhythmia [97]), with *I_Kr_* [33,34], *I_Ks_* [34], and *I_Na_* [98] inhibited by IL-6, altogether leading to the prolongation of APD [33,34,99]. Mechanistically, IL-6 trans-signaling may alter major ventricular ion channel function via (1) the defective trafficking of channel protein subunits to the heart cell surface, (2) abnormal gating properties of surface channels, and (3) multiple and diverse combinations of the above, highlighting the importance of multi-ion channel analyses that may inform the rational development of safer (reduced cardiotoxic effects) anti-arrhythmic monotherapy and polytherapy approaches for dangerous arrhythmias in patients. 

Furthermore, cardiac Purkinje cells (PCs), which form an extensive specialized conduction system with ventricular myocytes to initiate contraction, are important triggers of VT [100,101,102] and are frequently used in the pharmaceutical industry to evaluate the cardiac electrophysiology effects of QT-prolonging drugs [103,104]. While PC electrophysiology is known to differ from that of ventricular myocytes in regard to AP shape, intrinsically long APD [105,106], types and balance of membrane currents [107,108], the preferential development of EADs [109,110,111,112], and the ability of *I_Kr_* and *I_K_*_1_ inhibition to significantly lengthen repolarization to a greater degree in PCs than in ventricular muscle [113], no mechanistic data have been presented in support of a role for PCs in obesity-linked VT risk. Thus, the molecular basis for the potential PC bias in obesity-linked VT is unknown. 

Despite the advantages of selectively investigating a role for IL-6 trans-signaling in obesity-linked VT, other cytokines are also likely involved. Tumor necrosis factor alpha (TNF-α) has been shown to decrease *I_Kr_* [114], and the transient outward current is also inhibited by IL-1β [115] and IL-18 [116], while IL-1β [99] increases *I_Ca_*_,*L*_, altogether leading to prolongation of the APD. Therefore, the blockade of IL-6 in concert with other known molecular drivers of obesity-related ventricular arrhythmias may provide a more favorable clinical outcome than targeting IL-6 alone. For example, IL-1β or IL-18, both of which require the NLRP3 inflammasome for activation [117], can induce the production of copious amounts of IL-6, serving as an amplifier of IL-6′s effects. While the effects of IL-1β and TNF-α on cardiac electrophysiology have been studied, the role of IL-18 is under-explored. Because IL-18 has increased expression in obesity and HF [118,119,120] and contributes to heart rhythm disorders [121] and VT in mice [116], we suspect that IL-18 may heighten the IL-6 effects on ion channel function, pro-arrhythmic AP phenotypes, and ventricular arrhythmia risks. Although pro-inflammatory cytokine channelopathies play a role in the generation of arrhythmias, very little is known about the molecular mechanisms underlying their action. The identification of such mechanisms as a contributing factor for arrhythmias is likely to reveal novel targeted therapeutic avenues for the inflammatory system, including anti-inflammatory drugs, which may effectively help to prevent or reduce arrhythmic risk in patients.

## 4. Anti-IL-6 Inhibitors

Anti-IL-6 inhibitors are a class of FDA-approved drugs [122,123] that have been shown to confer major cardiovascular benefits [124], including reducing the risk for HF outcomes [125]. In that regard, IL-6-deficient mice have revealed that IL-6 contributes to the pathogenesis of many inflammatory diseases, including bacterial and viral infections, arthritis, experimental colitis, and multiple sclerosis [42,126]. Moreover, the selective inhibition of the IL-6 trans-signaling pathway, which mediates pro-inflammatory IL-6 effects in a rat model of re-perfused myocardial infarction, prevented IL-6 pro-inflammatory defects, reduced infarct size, and preserved cardiac function [127]. Furthermore, engineered extracellular vesicles expressing signaling-incompetent IL-6 signal transducer (ILST) decoy receptors that specifically blocked IL-6 trans-signaling in a Duchenne muscular dystrophy mouse model resulted in a reduction in STAT3 phosphorylation in the quadriceps and gastrocnemius muscles of the mice [128]. IL-6 knockout or neutralization prevented diet-induced inflammation [129], metabolic defects [129], and drug- and sterile pericarditis-linked arrhythmias [98,130]. Therefore, strategies to inhibit global IL-6 signaling pathways based on antibodies and small molecules that target IL-6, IL-6R, or the downstream signaling JAKs have shown beneficial cardiovascular effects when used as treatment options for chronic inflammatory diseases, COVID-19, and life-threatening cytokine storms associated with CAR T cell therapy in patients with cancer [122,131]. Taken together, these findings support the possibility that direct inhibition of the IL-6 signaling axis offers the potential to bridge such activities, one of the primary measures of success in heart failure-specific, especially arrhythmia-specific, therapy in obesity. 

TCZ, an IL-6R antibody (Ab) that globally blocks IL-6 activities through IL-6R-α and sIL-6R, is associated with a significant reduction in the QT_c_ interval in patients with rheumatoid arthritis (RA) [91]. This was of particular interest to us, as we had previously reported that the blockade of IL-6R with an inhibitory mouse monoclonal anti-IL-6R-α Ab prevented decreases in *I_Kr_* density due to IL-6 being present in guinea pig ventricular myocytes [33]. Moreover, TCZ has been recently shown to prevent electrophysiological abnormalities in IL-6-challenged guinea pigs [98]. However, the implications of targeting IL-6R for the prevention of ventricular arrhythmias in metabolic disorders are unknown. A lack of progress may be due to the risk of serious side effects of the current therapeutics used in chronic inflammatory diseases, particularly the suppression of not only the deleterious effects of the IL-6 pathway but also the beneficial IL-6 functions mediated by classical signaling. TCZ [50,98,132], when used in treating RA, is also associated with adverse cardiovascular events [133] and an increase in serum cholesterol [134], which likely enhances cardiovascular risk [135,136]. In this regard, effective therapeutic interventions for the prevention of arrhythmia/SCD in obesity would likely require chronic administration, which would render TCZ ineligible for the long-term treatment of chronic metabolic diseases and highlight the mechanistic relevance of selectively targeting IL-6 trans-signaling. 

The first natural and specific inhibitor of IL-6 trans-signaling, olamkicept, has shown encouraging results in phase II clinical studies for inflammatory bowel disease [42,137,138,139,140]. Although the selective blockade of IL-6 trans-signaling with sgp130Fc is known to attenuate atrial fibrillation (AF) inducibility in transverse aortic constriction-challenged mice [141], the potential therapeutic benefits and efficacy of selectively targeting IL-6 trans-signaling (olamkicept) for the prevention of ventricular arrhythmias, particularly in obesity, are unknown, which further provides the rationale to refine olamkicept for the development of the next generation of inhibitors of IL-6 trans-signaling as an anti-arrhythmic target. Moreover, the same obesity pathways that connect IL-6 trans-signaling to ventricular arrhythmias are likely involved in the adverse remodeling associated with other pathologies, including myocardial infarction [127,142] and HF [143]. We have recently [144] discovered in a guinea pig heart model that hyper-IL-6 (hIL-6) alone profoundly prolongs the QT interval and directly triggers arrhythmias. We further found that the guinea pigs challenged with a combined IL-6-sIL-6R had a prolonged QT interval, which can be prevented with olamkicept, highlighting an important anti-arrhythmic role for anti-IL-6 trans-signaling inhibitors. Thus, understanding the link between obesity, IL-6 trans-signaling, and ventricular arrhythmias may have broad clinical implications for many heart diseases. 

Crucially, IL-6 inhibition has the potential to enhance the efficacy and even exceed the beneficial effects (anti-arrhythmic) of anti-inflammatory drugs currently used in clinical trials. Intriguingly, while anti-inflammatory drugs (including non-steroidal anti-inflammatory drugs [145,146] and corticosteroids [147,148]) have shown beneficial anti-arrhythmic properties in experimental AF [149,150,151], all have shown an increased risk of AF in patients, and the role of these drugs in ventricular arrhythmias is not well established [150], likely because our knowledge of cytokine mechanisms in the ventricular inflammatory process is incomplete. 

## 5. Importance of Preclinical Models for Studying IL-6 Trans-Signaling

Popularly used rats and mice are the predominant models of human metabolism, inflammation, and ventricular arrhythmias. However, these models display low levels of total and low-density lipoprotein cholesterol, which significantly differ from humans. Compared with humans, mice have notable differences regarding their immune systems [152,153], which may limit the transferability of findings obtained in mouse models to a clinical setting in humans. Thus, preclinical models in larger animals are necessary to validate the findings obtained in mouse models prior to clinical application. Guinea pigs are one such model. Intriguingly, guinea pigs display appetite hormones [154], circulating cholesterol [155], a large fat storage capacity [156], and an inflammatory response [157] similar to humans, indicating that they are suitable for HFD-induced inflammation mechanistic studies [157,158]. Increasing numbers of investigators now use guinea pigs as models of arrhythmias [159,160,161,162], which further underscores their suitability. Moreover, the electrophysiology of a guinea pig heart is more like that of humans, whereas mice and rats display different action potential configurations, shorter refractory periods, greater densities of repolarizing K currents [163], and virtually absent *I_Kr_* and *I_Ks_* [164]. Because of the complexity of genetically manipulating guinea pigs, in addition to the experimental cost and duration of time required to develop high-fat diet-induced obesity arrhythmia models, using guinea pigs to routinely investigate the obesity-related pro-inflammatory mechanisms in the heart may be impractical. However, Golden Syrian hamsters represent a preclinical animal model that is relatively inexpensive and versatile, which may allow for the investigation of obesity and adverse ventricular remodeling mechanisms in the heart and myocytes. Golden Syrian hamsters are obesity-prone, develop insulin resistance, and, unlike rats and mice, develop hypercholesterolemia and hypertriglyceridemia when fed a cholesterol-rich diet [165,166]. The Golden Syrian hamster has also previously been used widely in studies of lipoprotein metabolism [167]. Importantly, hamsters express *I_K_* subunits [168,169], display major ventricular ionic currents, and trigger ventricular arrhythmias [170]. Collectively, these features show that the hamster may be an excellent species for obesity-related ventricular arrhythmia studies. Considering that the hamster recapitulates the electrical properties of human myocardia, future studies that utilize the Golden Syrian hamster are likely to advance the arrhythmia field by providing novel cellular pro-arrhythmic mechanisms to better understand unique phenotypes in patients and to predict arrhythmia penetrance. 

## 6. Insights into Significant Knowledge Gaps and Future Directions in Obesity, Inflammation, and Ventricular Arrhythmias

Evidence that anti-cytokine therapy might provide a valuable therapeutic adjunct for heart disease, particularly for the prevention of arrhythmias/SCD, is increasing in the extant literature. However, there is a paucity of mechanism-based pathological IL-6 trans-signaling processes in ventricular arrhythmia studies. Therefore, future efforts are required to test whether the therapeutic manipulation of IL-6 trans-signaling efficiently prevents pro-arrhythmic signatures (ion channel function, APD phenotypes, QT prolongation, and ventricular arrhythmia vulnerability) in diseased ventricular tissues/myocytes, thus counteracting (a) the lack of tissue specificity in current anti-cytokine treatments, (b) the redundancy of cytokines, and (c) the undesired side effects elicited by breaching the essential homeostatic role of classical IL-6 signaling. Such paradigms call for fresh perspectives gained through the meticulous molecular characterization of immune responses in disease traits that take into account the cell/tissue and signaling pathway specificity. 

This objective can be further advanced by asking several critical questions. For example, what are the unique electrophysiological features (including ECG phenotypes and cellular channel biophysics) of IL-6 trans-signaling-linked ventricular arrhythmias? What are the other key cellular pro-arrhythmic electrical mechanisms (*I_Ks_*, *I_Na_*, *I_Ca_*_,*L*_, *I_K_*_1_, and Ca handling) targeted by the IL-6 trans-signaling pathway, and how might they promote arrhythmia/SCD risk? Can we genetically or pharmacologically inhibit IL-6 trans-signaling and prevent arrhythmia? What are the key IL-6 trans-signaling molecular partners, and can we identify novel “druggable/actionable” targets? Might existing drugs (including olamkicept or sodium–glucose cotransporter 2 (SGLT-2) inhibitors) prove useful as anti-arrhythmic drugs if we can demonstrate that specific targets might be key mediators of obesity-related malignant VT? What is the functional outcome of targeting IL-6 trans-signaling in the presence of other cytokines (IL-1β, TNF-α, and IL-18) in obesity? What are the direct actions of IL-6 trans-signaling on working myocytes, and why are the involved cellular mechanisms less understood, particularly with regard to their electrophysiology and second messenger pathways? 

**Figure 3:**
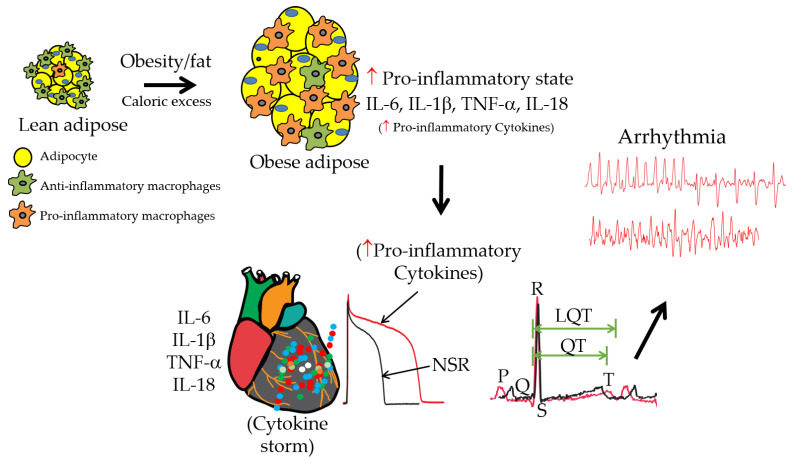
Cartoon illustration shows the hypothesized pro-inflammatory cytokine-linked channelopathies leading to pathological APD prolongation and ventricular arrhythmias. Obesity or excessive eating is associated with enlarged adipose tissue, leading to increased levels of a heightened pro-inflammatory response defined by a cytokine storm of overactivation of pro-inflammatory cytokines (IL-6, IL-1β, TNF-α, and IL-18), all of which are likely to play crucial roles in adverse remodeling of the relative functional expression of major ventricular repolarizing (*I_Kr_* and *I_Ks_*) and depolarizing (*I_Ca_* and *I_Na_*) macroscopic currents leading to delayed repolarization, which increases the risk for fatal malignant ventricular arrhythmias with obesity. Furthermore, macrophages and adipocytes infiltrate the heart at the onset of metabolic and inflammatory disorders and contribute to cardiac dysfunction, possibly through pathological local levels of individual and multiple combinations of pro-inflammatory cytokines.

Epicardial adipose tissue (EAT, Figure 3) is a rich local source of IL-6 signaling in obesity [171,172] and a key contributor to a higher risk of cardiac events in patients [173,174]. In this context, Ernault et al. previously showed that atrial EAT-derived secretomes facilitate sustained reentrant arrhythmias when compared with subcutaneous adipose tissue secretomes or cardiomyocyte-conditioned media [175] and that this occurs through the decreased expression of *I_K_*_1_ and connexin-43. These findings predict that EAT-derived cytokine mechanisms are likely to adequately mirror local IL-6 trans-signaling-defined pathophysiological conditions and reveal prime candidates for augmented cellular ventricular arrhythmogenesis and increased SCD risk with obesity.

Furthermore, macrophages are associated with heightened immune responses to infectious pathogens and tissue damage in obesity [176], while enlarged adipocytes release saturated FFAs through lipolysis [177]. In this regard, the chronic accumulation of lipid droplets within the myocardium will be expected to promote infiltration of activated macrophages and epicardial adipocytes, resulting in heightened IL-6 trans-signaling and warranting further investigation. One way to achieve this is via in silico models. Computational modeling will accomplish two goals: (1) the creation of cellular models that provide a physiological understanding of individual or multiple combinations of cytokines’ pro-arrhythmic effects; and (2) the integration of cell models into ventricular tissue 2D or 3D models to predict tissue-level mechanisms of arrhythmia, including the subcellular incidence of alternans, afterdepolarizations, the formation of triggered beats, and changes in conduction velocity, as well as a vulnerability window of unidirectional conduction block and arrhythmias. Another important in vitro model is cultured cells differentiated into cardiomyocytes from human-induced pluripotent cells (hiPSC-CMs), which have been very useful in understanding the cellular phenotypic characteristics of cardiomyocytes and in comparing these cellular phenotypes with normal individuals and obese patients with heart disease [178,179]. This type of human heart modeling increasingly constitutes an important preclinical human model or cellular complement in studying arrhythmia mechanisms in the intact hearts of model animals.

## 7. Conclusions

Obesity is a multifactorial chronic disease where the initiated signaling cascades are complex, and other pathways could be involved in the increased arrhythmia risk. Although focusing on the pathological role of how an individual obesity mediator predisposes to arrhythmia is the typical approach, conceptually, the complexity of obesity suggests that the individual and synergistic molecular mechanisms of cytokines/ion channels/cardiomyocytes likely limit a holistic understanding of the functional coupling between obesity and arrhythmias. Although significant advances have been made in prognostic approaches and anti-arrhythmic drugs, significant adjustments to the existing approaches that will lead to deeper mechanistic insights are still clearly needed. Despite these complexities, an important goal is to continue to make novel discoveries in established preclinical models that will help translate our work into preclinical trials with large animals, human tissue/myocyte models, and, ultimately, humans. Without such information, the promise of a novel class of anti-IL-6 therapies for the treatment of human ventricular arrhythmia will likely remain unfulfilled.

## Data Availability

All of the relevant data are included in this paper.

## Abbreviations

**Nonstandard Abbreviations and Acronyms**	
TCZ	Tocilizumab
*I* *_Kr_*	Rapidly activating delayed rectifier K current
*I* *_Ks_*	Slowly activating delayed rectifier K current
*I* *_Na_*	Sodium current
*I*_Ca_,*_L_*	L-type Ca current
*I* *_K_*_1_	Inwardly rectifying K current
LQT	Long QT
QT_c_	QT interval corrected for heart rate
FFAs	Free fatty acids
AF	Atrial fibrillation
hERG	Human ether-á-go-go-related gene
HEK	Human embryonic kidney
ERG	Ether-á-go-go-related gene
APD	Action potential duration
EAT	Epicardial adipose tissue
SCD	Sudden cardiac death
VF	Ventricular fibrillation
VT	Ventricular tachycardia
PC	Purkinje cells
